# Intrinsic Computation of a Monod-Wyman-Changeux Molecule

**DOI:** 10.3390/e20080599

**Published:** 2018-08-11

**Authors:** Sarah Marzen

**Affiliations:** Physics of Living Systems Group, Department of Physics, Massachusetts Institute of Technology, Cambridge, MA 02139, USA; semarzen@mit.edu

**Keywords:** statistical complexity, intrinsic computation, excess entropy

## Abstract

Causal states are minimal sufficient statistics of prediction of a stochastic process, their coding cost is called statistical complexity, and the implied causal structure yields a sense of the process’ “intrinsic computation”. We discuss how statistical complexity changes with slight changes to the underlying model– in this case, a biologically-motivated dynamical model, that of a Monod-Wyman-Changeux molecule. Perturbations to kinetic rates cause statistical complexity to jump from finite to infinite. The same is not true for excess entropy, the mutual information between past and future, or for the molecule’s transfer function. We discuss the implications of this for the relationship between intrinsic and functional computation of biological sensory systems.

## 1. Introduction

Intrinsic computation [1] is a theory of how a dynamical system “intrinsically” computes. In short, one makes a minimal maximally predictive model (or ϵ-machine) of the process generated by a dynamical system. States of the ϵ-machine are called “causal states”, although these states are normally not causal in the sense of Ref. [2]. Certain words are forbidden, in that those words can never be seen. The words that are seen and thus accepted by the ϵ-machine constitute the ϵ-machine’s language, in a nod to the computation performable by finite and infinite automata. The “memory stored by the process”, the statistical complexity, is taken to mean the coding cost of the ϵ-machine’s states.

One interesting hypothesis is that the ϵ-machine’s structure provides a guide to the “functional” computation of the corresponding dynamical system. Functional computation–biologically-relevant computation, e.g., transformation of information with fitness consequences–might include everything from estimating past input [3,4] to predicting future input [5] to performing logical computations on input [6]. A more rigorous definition of “functional computation” remains an open problem; here, we merely list examples of quantities that can be identified with functional computations. As of yet, no link between intrinsic and functional computation has been found.

Here, we investigate the intrinsic computation and two functional computations (ligand concentration transduction and low-pass filtering) of a Monod-Wyman-Changeux (MWC) molecule, a widely-used model of a biological sensor [7,8,9]. This is the first time that the intrinsic computation of an MWC molecule–which here is limited to the ϵ-machine structure, the statistical complexity, and the excess entropy–has been calculated. The calculational techniques used here can be applied to study intrinsic computation of a more general class of biological sensors than previously studied.

We find that certain arbitrarily small perturbations to the underlying MWC molecule can lead to arbitrarily large perturbations in the process’ intrinsic structure but lead to arbitrarily small changes in the stated functional computations. To the author’s knowledge, this is the first such example in the literature. These results therefore suggest that causal structure and functional computation are orthogonal characterizations of a process, at least for oft-considered functional computations.

However, intrinsic computation could be taken to include several newer structure-related information-theoretic measures of a process, including excess entropy [10,11,12]. These newer measures do not suffer the same sensitivity as the ϵ-machine and statistical complexity, suggesting that these measures might help characterize functional computation.

Section 2 reviews the definition of ϵ-machine and MWC molecules. Section 3 explores how variations in kinetic rates change the molecule’s intrinsic and functional computation. Section 4 discusses future research directions for intrinsic computation.

## 2. Background

The subject of interest here is a continuous-time, discrete-event process. However, for reasons explained later, for characterization of statistical complexity we will consider time-binning the process and treating it as a discrete-time, discrete-event process. Hence, we are also interested in discrete-time, discrete-event processes.

First, we discuss discrete-time, discrete-event processes. We code these processes as $\ldots ,x_{-1},x_{0},x_{1},\ldots$ where $x_{i}$ is the *i*-th symbol appearing. The past $\overset{\leftarrow }{x}$ (with corresponding random variable $\overset{\leftarrow }{X}$) is taken to be $\ldots ,x_{-2},x_{-1}$ while the future $\vec{x}$ (with corresponding random variable $\vec{X}$) is taken to be $x_{0},x_{1},x_{2},\ldots$.

Next, we discuss continuous-time, discrete-event processes. We code these processes as $\ldots ,\left(x_{-1},\tau_{-1}\right),\left(x_{0},\tau_{0}\right),\left(x_{1},\tau_{1}\right),\ldots$ where $x_{i}$ is the *i*-th symbol, appearing for a total duration $\tau_{i}$. We enforce $x_{i}\neq x_{i+1}$ so as to ensure a unique coding. The present is said to occur sometime during the presentation of $x_{0}$, and so we denote the past $\overset{\leftarrow }{\left(x,\tau \right)}$ (with corresponding random variable $\overset{\leftarrow }{\left(X,T\right)}$) as $\ldots ,\left(x_{-1},\tau_{-1}\right),\left(x_{0},\tau_{+}\right)$ and the future $\vec{\left(x,\tau \right)}$ (with corresponding random variable $\vec{\left(X,T\right)}$) as $\left(x_{0},\tau_{-}\right),\left(x_{1},\tau_{1}\right),\ldots$ where $\tau_{+}+\tau_{-}=\tau_{0}$.

Section 2.1 reviews the definition of causal states, statistical complexity, the continuous-time ϵ-machine, and the mixed-state simplex. Section 2.2 reviews the dynamical models of Monod-Wyman-Changeux molecules used here.

We assume knowledge of information theory at the level of Ref. [13], but we briefly review definitions here. When *X* is a discrete random variable with probability distribution p(x), then its entropy is $H\left[X\right]=-\sum_{x}p\left(x\right)\log p\left(x\right)$; when *X* is a continuous random variable with probability density function ρ(x), then the differential entropy is H[X]=−∫ρ(x)logρ(x)dx; and when *X* is a mixed random variable (as is the case here), the entropy H[X] is given by Ref. [14]. Entropy can be thought of as a measure of uncertainty. Conditional entropy of *X* conditioned on random variable *Y* is $H\left[X|Y\right]={\langle H\left[X|Y=y\right]\rangle }_{y}$, and the mutual or shared information I[X;Y] between two random variables *X* and *Y* is merely I[X;Y]=H[X]−H[X|Y].

### 2.1. Causal States S, Statistical Complexity $C_{\mu }$, the ϵ-Machine, and the Mixed-State Simplex

Consider the equivalence relation ${∼}_{\epsilon }$ that clusters two semi-infinite pasts, $\overset{\leftarrow }{x}$ and ${\overset{\leftarrow }{x}}^{\prime }$, together if $\mathrm{Pr}\left(\vec{X}|\overset{\leftarrow }{X}=\overset{\leftarrow }{x}\right)=\mathrm{Pr}\left(\vec{X}|\overset{\leftarrow }{X}={\overset{\leftarrow }{x}}^{\prime }\right)$–that is, if the two pasts are equivalent from the standpoint of prediction. The corresponding clusters are causal states σ, which are realizations of the random variable for the causal states S. The statistical complexity $C_{\mu }$ is simply their coding cost, $C_{\mu }=H\left[S\right]$. In short, causal states S are minimal sufficient statistics of prediction; the statistical complexity $C_{\mu }=H\left[S\right]$ is the coding cost of those causal states [15]; and the ϵ-machine is the minimal maximally predictive model constructed from those causal states [16].

The same constructions apply when considering continuous-time, discrete-event processes. In that case, the equivalence relation ${∼}_{\epsilon }$ clusters two semi-infinite pasts $\overset{\leftarrow }{\left(x,\tau \right)},\;{\overset{\leftarrow }{\left(x,\tau \right)}}^{\prime }$ together if $\mathrm{Pr}\left(\vec{\left(X,T\right)}|\overset{\leftarrow }{\left(X,T\right)}=\overset{\leftarrow }{\left(x,\tau \right)}\right)=\mathrm{Pr}\left(\vec{\left(X,T\right)}|\overset{\leftarrow }{\left(X,T\right)}={\overset{\leftarrow }{\left(x,\tau \right)}}^{\prime }\right)$. Again, the clusters are causal states σ, realizations of the random variable for causal states S.

For what follows, we must define two terms: mixed state simplex, and unifilar. Consider any hidden Markov model whose hidden state at time *t* is a random variable; now consider the probability distribution over hidden states given observations $\overset{\leftarrow }{x}$. Each one of these conditional probability distributions is a mixed state, and it lies in the mixed state simplex, the set of all possible probability distributions over hidden states. The hidden Markov model is unifilar when, given one’s hidden state at time *t* and an observation at time *t*, one knows exactly which hidden state comes next at t+1. There is a connection between unifilarity and the mixed state simplex: when the hidden Markov model under study is unifilar, then the mixed states will lie at the edge of the simplex. (This is not true for nonunifilar hidden Markov models.) The causal states are just the mixed states of the minimal (potentially nonunifilar) generative model.

The causal states of discrete-time processes are usually uncountably infinite. When this is the case, then the box-counting dimension of the mixed state presentation in the mixed state simplex is nonzero. Let’s unpack this statement. Suppose that a (potentially nonunifilar) Hidden Markov model with states *g* generates the observed discrete-time process. Then we use $p(g|\overset{\leftarrow }{x})$ to denote the probability over hidden states in the generative model given past output. Typically, $p(g|\overset{\leftarrow }{x})$ is in the interior of the mixed state simplex–the space of probability distributions over hidden states. The box-counting dimension of the mixed state presentation is obtained by gridding the mixed state simplex by cubes of side length ϵ, counting the number of non-empty cubes $N_{\epsilon }$ (which contain coarse-grainings of histories) [17], and then calculating the scaling of $N_{\epsilon }$ with ϵ–so the box-counting dimension is $\lim_{\epsilon \to 0}\frac{\log N_{\epsilon }}{\log\frac{1}{\epsilon }}$. A cube is considered non-empty when there is at least one history that leads to a mixed state in the cube. When there are countable causal states for a discrete-time process, the box-counting dimension is 0.

The causal states inherit a dynamic, and the ϵ-machine of a process is the pairing of causal states together with that dynamic. For discrete-time, discrete-event processes, tractable ϵ-machines are merely countable unifilar Hidden Markov models [16], where unifilarity implies that the next hidden state is determined uniquely by the previous hidden state and the present emitted symbol. For continuous-time, discrete-event processes, tractable ϵ-machines can (for instance) take the form of joined conveyer belts [18]. Continuous-time causal states are then usually accompanied by labeled transition operators $O^{\left(x\right)}$, and the list of labeled transition operators specifies the continuous-time ϵ-machine. A “tractable ϵ-machine” is one for which $C_{\mu }$ is finite, with the exception of the ϵ-machines of continuous-time periodic processes (which are tractable but which correspond to infinite $C_{\mu }$).

### 2.2. Monod-Wyman-Changeux Molecules

**Figure 1:**
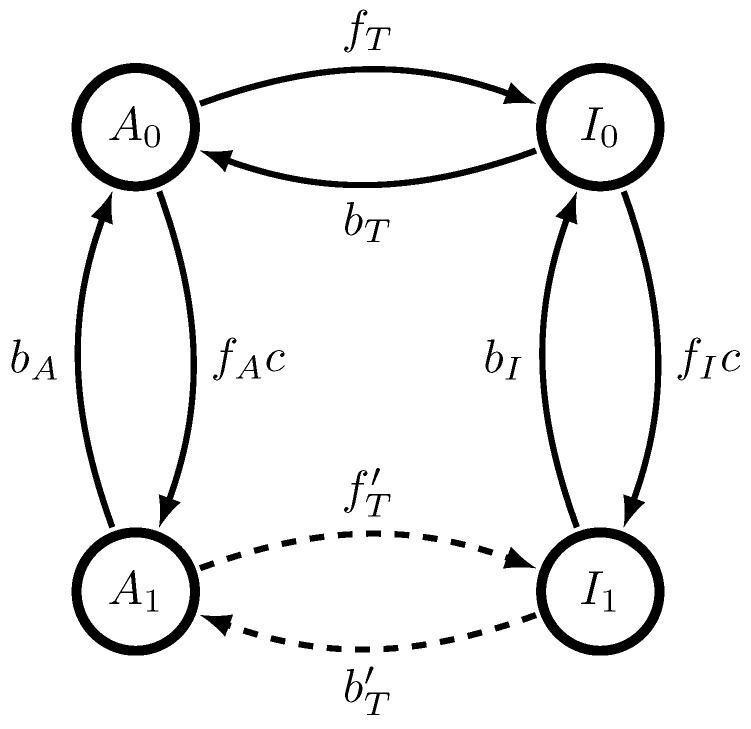
A dynamical single-site Monod-Wyman-Changeux molecule, with kinetic rates as shown. States marked $A_{i}$ are active with *i* bound ligand molecules, while states marked $I_{i}$ are inactive with *i* bound ligand molecules. When transitioning from states $A_{0},\;A_{1}$, *A* is emitted, while when transitioning from states $I_{0},\;I_{1}$, *I* is emitted.

A Monod-Wyman-Changeux (MWC) molecule has two configurations, active (A) and inactive (I), and *n* binding sites. Each configuration can bind any number of molecules, from 0 to *n*. This gives a total of $2^{n+1}$ possible states. If binding sites are indistinguishable, then a simplified model can be made based on the symmetry in binding sites so that there are only 2(n+1) distinguishable possible states: it can be either active or inactive, with any number of binding sites occupied by ligand molecules. As our argument holds for any *n*, we focus on the case that n=1. The four states of the corresponding MWC molecule–$\left\{A_{0},A_{1},I_{0},I_{1}\right\}$, standing for active/inactive (A/I) with either 0 or 1 ligands bound as written in the subscript–are shown in Figure 1, along with allowed transitions.

We denote the probability distribution of being in various states as

(1) $$\vec{p}=\left(\begin{array}{c} p(A_{0})\\ p(A_{1})\\ p(I_{0})\\ p(I_{1}) \end{array}\right).$$

This probability distribution evolves via the master equation

(2) $$\frac{d\vec{p}}{dt}=M\left(c\left(t\right)\right)\vec{p}$$

where

(3) $$M\left(c\right)=\left(\begin{array}{cccc} -(f_{T}+f_{A}c) & b_{A} & b_{T} & 0\\ f_{A}c & -(f_{T}^{\prime }+b_{A}) & 0 & b_{T}^{\prime }\\ f_{T} & 0 & -(b_{T}+f_{I}c) & b_{I}\\ 0 & f_{T}^{\prime } & f_{I}c & -(b_{T}^{\prime }+b_{I}) \end{array}\right)$$

with $f_{T},\;f_{A},\;b_{A},\;b_{T},\;f_{T}^{\prime },\;b_{T}^{\prime }$ are kinetic parameters. In fixed ligand concentration *c*, Equation (2) is solved as

(4) $$\vec{p}\left(t\right)=e^{M(c)t}\vec{p}\left(0\right)$$

where $\vec{p}\left(0\right)$ is the initial probability distribution over the MWC molecule’s states.

## 3. Results

We suppose that we are only allowed to see whether or not the MWC molecule is active or inactive, as would be true for most experimental observations of ligand-gated ion channels. This is a key constraint, as otherwise, the minimal generative model would be the minimal maximally predictive model and none of the discrepancies described here would arise. In what follows, we explore the effects of kinetic rates on intrinsic and functional computation.

Intrinsic computation as it was originally defined included the ϵ-machine and statistical complexity, and today includes other information measures, such as the excess entropy [10,11,12]. The number of functional computation-related quantities is unbounded, but we focus on two here due to their presence in the literature: the binding curve, or the probability of the MWC molecule being active as a function of ligand concentration; and the transfer function, or how the MWC molecule responds to sinusoidal perturbations of the ligand concentration.

Our argument will essentially be a proof by contradiction. We will start by assuming that there *is* some relationship between at least one aspect of intrinsic computation and at least one aspect of functional computation. If there were a relationship between these two quantities, then we should not be able to change kinetic rates so that one quantity changes by an arbitrarily small amount and the other by an arbitrarily large amount. (If so, these kinetic rates would then be of incredible importance to the process’ causal architecture, say, but of vanishingly small importance to the so-called functional computations.) We will then show in the following analysis that arbitrarily small perturbations in the kinetic rates $f_{T}^{\prime },\;b_{T}^{\prime }$ induce arbitrarily large perturbations to the ϵ-machine and statistical complexity, but induce arbitrarily small perturbations to excess entropy and the functional computations considered here. We therefore conclude that if there is a relationship between intrinsic computation and functional computation for these kinds of molecules, it will more likely come from excess entropy (or other more recently-studied information measures of time series [19]) than from statistical complexity or the ϵ-machine. We discuss the possibilities of finding functional computations that are sensitive to arbitrarily small increases in $f_{T}^{\prime },\;b_{T}^{\prime }$ in Section 4.

### 3.1. Intrinsic Computation

If $b_{T}^{\prime },\;f_{T}^{\prime }>0$, then there is no “sync word”–that is, no string of observed past symbols that uniquely determines the underlying present state of the MWC molecule. (Note again that this would not be the case if we were allowed to observe the full state, and not just whether or not the MWC molecule is active or inactive.) This has important consequences for $C_{\mu }$ and for the process’ ϵ-machine.

To analyze $C_{\mu }$, we move to the discrete-time domain, so as to avoid the interpretational difficulties with differential entropy [18]. By observing the process every Δt for Δt much smaller than any inherent time constant in the problem, the process is turned into a discrete-time process. The transition probabilities of this new process have corresponding labeled transition matrices $T^{\left(x\right)}=e^{M^{\left(x\right)}\Delta t}$, which are approximated to lowest order in Δt by $T^{\left(x\right)}=I^{\left(x\right)}+M^{\left(x\right)}\Delta t$, where *x* is an emitted symbol:

$$\begin{array}{ccc} M^{\left(A\right)}\left(c\right) & = & \left(\begin{array}{cccc} -(f_{T}+f_{A}c) & b_{A} & 0 & 0\\ f_{A}c & -(b_{A}+f_{T}^{\prime }) & 0 & 0\\ f_{T} & 0 & 0 & 0\\ 0 & f_{T}^{\prime } & 0 & 0 \end{array}\right)\;\mathrm{and}\\ M^{\left(I\right)}\left(c\right) & = & \left(\begin{array}{cccc} 0 & 0 & b_{T} & 0\\ 0 & 0 & 0 & b_{T}^{\prime }\\ 0 & 0 & -(b_{T}+f_{I}c) & b_{I}\\ 0 & 0 & f_{I}c & -(b_{I}+b_{T}^{\prime }) \end{array}\right) \end{array}$$

and

$$\begin{array}{ccc} I^{\left(A\right)} & = & \left(\begin{array}{cc} I_{2\times 2} & 0_{2\times 2}\\ 0_{2\times 2} & 0_{2\times 2} \end{array}\right)\\ I^{\left(I\right)} & = & \left(\begin{array}{cc} 0_{2\times 2} & 0_{2\times 2}\\ 0_{2\times 2} & I_{2\times 2} \end{array}\right). \end{array}$$

The causal states correspond to the mixed states $\mathrm{Pr}(S|\overset{\leftarrow }{X}=\overset{\leftarrow }{x})$ defined by either (a,1−a,0,0) or (0,0,1−b,b). When $b_{T}^{\prime },\;f_{T}^{\prime }>0$, all *a*’s and *b*’s are allowed; there are an uncountable infinity of these causal states because there is no sync word. As such, the ϵ-machine is uncountable and intractable, and statistical complexity is very likely infinite. However, when $b_{T}^{\prime }=f_{T}^{\prime }=0$, then only a countable set of *a*’s and *b*’s are allowed according to the discrete-time analogue of Theorem 1 of Ref. [18]. In particular, the causal states are identified as both the present configuration (active or inactive) and the number of time steps since last configuration switch. As a result, the ϵ-machine is countably infinite and tractable, and statistical complexity is finite, though it increases with $\log\frac{1}{\Delta t}$ [20].

**Figure 2:**
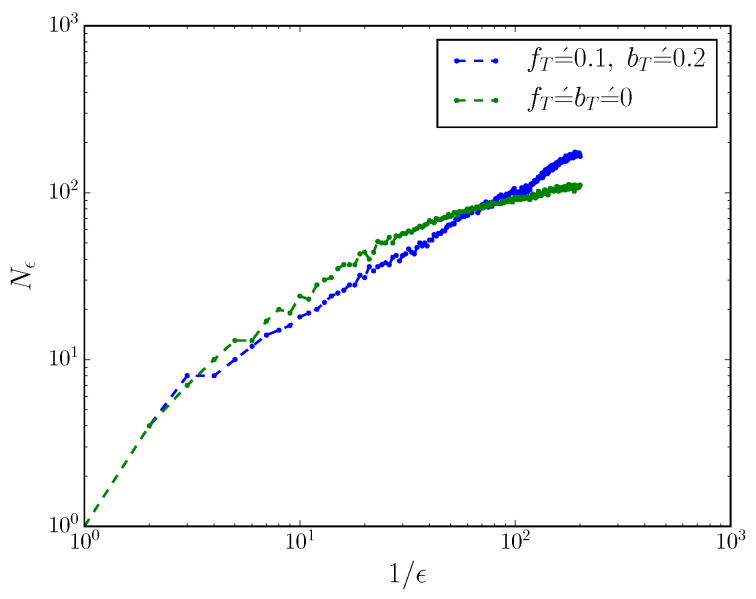
Box-counting dimension of the mixed-state presentation changes drastically with $f_{T}^{\prime },\;b_{T}^{\prime }$. For both processes, we have: $f_{T}=1.0,\;f_{A}c=2.9,\;b_{A}=3.4,\;b_{T}=3,\;f_{I}c=4,\;b_{I}=2$. The process with nonzero $f_{T}^{\prime },\;b_{T}^{\prime }$ has a scaling of $\log N_{\epsilon }∼\log\left(1/\epsilon \right)$ and thus a nonzero box-counting dimension $h_{0}>0$, whereas the process with $f_{T}^{\prime }=b_{T}^{\prime }=0$ has a scaling of $\log N_{\epsilon }∼\log\log\left(1/\epsilon \right)$ and thus a box-counting dimension $h_{0}=0$.

This can be seen more directly by considering the mixed-state presentation’s box-counting dimension $h_{0}$ when the process is turned into a discrete-time process with small time resolution Δt=0.01. We coarse-grain mixed-state simplex into cubes of side-length ϵ, and count the number of non-empty boxes $N_{\epsilon }$, as described in Ref. [17,21]. The scaling of $N_{\epsilon }$ with ϵ reveals the box-counting dimension $h_{0}$ of the mixed-state presentation, via $N_{\epsilon }∼{\left(1/\epsilon \right)}^{h_{0}}$. Figure 2 shows the scaling of $N_{\epsilon }$ with ϵ for an MWC molecule with and without $f_{T}^{\prime }=b_{T}^{\prime }=0$. When $f_{T}^{\prime }=b_{T}^{\prime }=0$, $h_{0}=0$; when $f_{T}^{\prime },\;b_{T}^{\prime }>0$, the box-counting dimension $h_{0}>0$.

**Figure 3:**
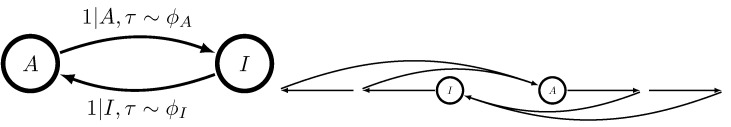
At left, a generative model of the process generated by the MWC molecule in fixed ligand concentration *c* of Figure 1 with $f_{T}^{\prime }=b_{T}^{\prime }=0$. The dwell time distributions $\phi_{A}\left(t\right)$ and $\phi_{I}\left(t\right)$ are given in Equations (8) and (9). At right, the corresponding topological ϵ-machine. While emitting *A*, one moves along the “conveyer belt” starting with state *A* to the left; while emitting *I*, one moves along the conveyer belt starting with state *I* to the right. To switch the letter that one is emitting, one jumps to the other conveyer belt. The states along the conveyer belt to the left correspond to the time that one has been inactive, and the states along the conveyer belt to the right correspond to the time that one has been active.

The reason for the former fact lies in Theorem 1 of Ref. [18]. When $f_{T}^{\prime }=b_{T}^{\prime }=0$, the dynamic MWC molecule of Figure 1 generates a semi-Markov process, a restricted version of the unifilar hidden semi-Markov processes analyzed in Ref. [18]. This is true even when there is more than one ligand binding site. Causal states are characterized by *x*, whether or not the MWC molecule is presently active, and $\tau_{+}$, the time since the MWC molecule last switched between activities. The now-tractable ϵ-machine takes the form shown in Figure 3.

In the continuous-time limit, which one can derive by considering the limit of the discrete-time process considered above with appropriate renormalization (e.g., compare Ref. [20] to Ref. [22]), all probability distributions over mixed states become probability density functions. Continuous-time statistical complexity can be defined using the entropy of mixed random variables [18,22], though differential entropy does have some troubling properties mentioned in those references. Given the analysis above, there is likely a singular limit in the continuous-time statistical complexity as the kinetic rates $b_{T}^{\prime },\;f_{T}^{\prime }$ tend to 0.

Not all structure-based characterizations of a process lack robustness in this way, as different structure-based metrics pick up on different kinds of structure. To show this, we now compare the statistical complexity $C_{\mu }$ and the excess entropy $E=I[\overset{\leftarrow }{\left(X,T\right)};\vec{\left(X,T\right)}]$ [10,11,12] when $f_{T}^{\prime }=\;b_{T}^{\prime }=0$. (We calculate E of the continuous-time process, as the excess entropy of the discrete-time process converges to that of the continuous-time process in the Δt→0 limit [20].) The latter can be calculated via $E=I[S^{+};S^{-}]$ [23,24], while $C_{\mu }=H\left[S^{+}\right]$, and so calculation of both merely requires the joint distribution $p(\sigma^{+},\sigma^{-})$. For that, we need $\phi_{A/I}\left(t\right)$, the dwell time distributions of activity and inactivity. Note that emission of an *A* implies that one has just landed in $A_{0}$, and similarly, emission of an *I* implies that one has just landed in $I_{0}$. Hence, $\phi_{A}\left(t\right)$ is the first-passage time distribution to state $I_{0}$ in which one starts in $A_{0}$; similarly, $\phi_{I}\left(t\right)$ is the first-passage time distribution to state $A_{0}$ in which one starts in $I_{0}$. To aid with the calculation, we recall the labeled transition matrices of Equation (5) when $f_{T}^{\prime }=b_{T}^{\prime }=0$. The matrix $M^{\left(A\right)}$ includes only the transitions between various active conformations and the only transition from active to inactive, $A_{0}\to I_{0}$. Therefore, the probability of not having stayed in active states given that one started in $A_{0}$ after a time *t* is given by

(5) $$1-\Phi_{A}\left(t\right)={\left({\hat{e}}_{3}+{\hat{e}}_{4}\right)}^{⊤}\vec{p}\left(t\right),\;\frac{d\vec{p}}{dt}=M^{\left(A\right)}\left(c\right)\vec{p}\left(t\right),\;\vec{p}\left(0\right)={\hat{e}}_{1},$$

where ${\hat{e}}_{k}$ is the vector with elements $\delta_{i,k}$. Hence, the survival function $\Phi_{A}\left(\tau \right)=\int_{\tau }^{\infty }\phi_{A}\left(\tau^{\prime }\right)d\tau^{\prime }$, the probability that one stays in the active conformation (one of $A_{0},\;A_{1}$) after time *t* given that one started in $A_{0}$, can be calculated via

(6) $$\Phi_{A}\left(t\right)=1-{\left({\hat{e}}_{3}+{\hat{e}}_{4}\right)}^{⊤}e^{M^{\left(A\right)}\left(c\right)t}{\hat{e}}_{1}.$$

Similarly,

(7) $$\Phi_{I}\left(t\right)=1-{\left({\hat{e}}_{1}+{\hat{e}}_{2}\right)}^{⊤}e^{M^{\left(I\right)}\left(c\right)t}{\hat{e}}_{3}.$$

After differentiation, we find that

(8) $$\begin{array}{ccc} \phi_{A}\left(t\right) & = & -\frac{d\Phi_{A}\left(t\right)}{dt}\\ & = & {\left({\hat{e}}_{3}+{\hat{e}}_{4}\right)}^{⊤}M^{\left(A\right)}\left(c\right)e^{M^{\left(A\right)}\left(c\right)t}{\hat{e}}_{1} \end{array}$$

(9) $$\begin{array}{ccc} \phi_{I}\left(t\right) & = & -\frac{d\Phi_{I}\left(t\right)}{dt}\\ & = & {\left({\hat{e}}_{1}+{\hat{e}}_{2}\right)}^{⊤}M^{\left(I\right)}\left(c\right)e^{M^{\left(I\right)}\left(c\right)t}{\hat{e}}_{3}. \end{array}$$

**Figure 4:**
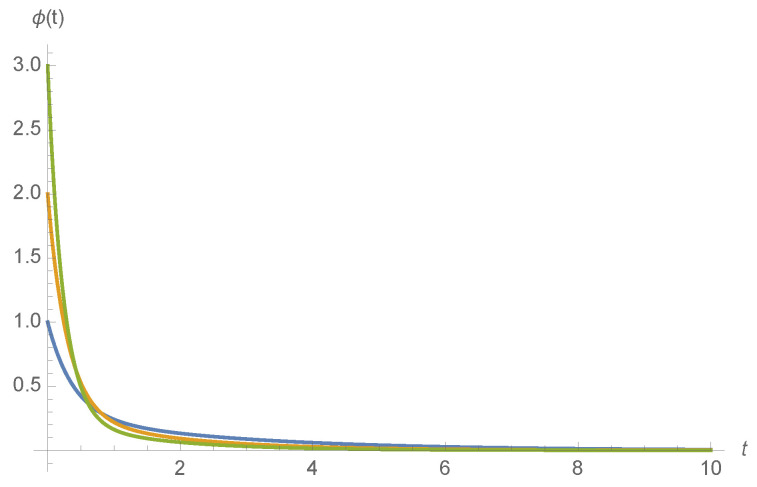
$\phi_{A}\left(t\right)$ for $f_{A}=b_{A}=1.0$ and $f_{T}=1.0$ (blue), $f_{T}=2.0$ (orange), and $f_{T}=3.0$ (green), calculated using Equation (8).

Examples of $\phi_{A}\left(t\right)$ for various ligand concentrations *c* and kinetic rates are shown in Figure 4.

**Figure 5:**
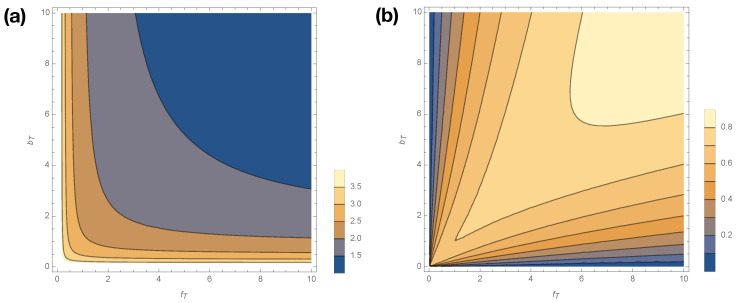
Contour plot of $C_{\mu }$ (**a**) and E (**b**) as a function of $f_{T},\;b_{T}$ when $f_{T}^{\prime }=b_{T}^{\prime }=0,\;f_{A}c=f_{I}c=b_{A}=b_{I}=1$.

From Lemma 1 of Ref. [18], we find that the statistical complexity of this semi-Markov process is given by

(10) $$C_{\mu }=H_{b}\left(p\left(A\right)\right)-\sum_{x\in \{A,I\}}p\left(x\right)\int_{0}^{\infty }\left(\mu_{x}\Phi_{x}\left(\tau \right)\right)\log\left(\mu_{x}\Phi_{x}\left(\tau \right)\right)d\tau ,$$

where $p\left(A\right)=\frac{\mu_{I}}{\mu_{A}+\mu_{I}}$, $\mu_{x}=1/\int_{0}^{\infty }\Phi_{x}\left(\tau \right)d\tau$, and $H_{b}\left(x\right):=-x\log x-\left(1-x\right)\log\left(1-x\right)$. Figure 5 shows how $C_{\mu }$ smoothly varies with changes in $f_{T},\;b_{T}$ for $f_{T},\;b_{T}>0$. Statistical complexity is maximized at small kinetic rates, $f_{T},\;b_{T}\to 0$; when those kinetic rates are small, dwell time distributions have longer tails, and the memory required to losslessly predict increases. Interestingly, if either $f_{T}$ or $b_{T}$ is exactly 0, then the generated process emits only *A* or *I* (see Figure 1) and thus has $C_{\mu }=0$. In other words, the limits $f_{T},\;b_{T}\to 0$ are singular, as were the limits $f_{T}^{\prime },\;b_{T}^{\prime }\to 0$.

We now wish to calculate $E=I[\overset{\leftarrow }{\left(X,T\right)};\vec{\left(X,T\right)}]$ which is $E=I[S^{+};S^{-}]$ [23], and so

(11) $$E=H\left[S^{-}\right]-H\left[S^{-}|S^{+}\right].$$

As a semi-Markov process is causally reversible, we have

(12) $$H\left[S^{-}\right]=H\left[S^{+}\right]=C_{\mu }$$

as given in Equation (10). Furthermore, the reverse-time causal states are the pair $\left(x_{-},\tau_{-}\right)$ (the time to next symbol and present symbol) while the forward-time causal states are still the pair $\left(x_{+},\tau_{+}\right)$ (the time since last symbol and present symbol) [18], so that $x_{+}=x_{-}$ almost surely, implying that $H[X_{-}|X_{+}]=0$. Hence,

(13) $$\begin{array}{ccc} H[S^{-}|S^{+}] & = & H[X_{-},T_{-}|X_{+},T_{+}]\\ & = & H\left[X_{-}|X_{+},T_{+}\right]+H\left[T_{-}|X_{-},X_{+},T_{+}\right]\\ & = & H[T_{-}|X_{0},T_{+}] \end{array}$$

where $x_{0}$ is just the present symbol. We then note that

(14) $$p\left(\tau_{-}|x_{0},\tau_{+}\right)=\frac{\phi_{x_{0}}\left(\tau_{+}+\tau_{-}\right)}{\Phi_{x_{0}}\left(\tau_{+}\right)},$$

as was derived in Ref. [22] for a continuous-time renewal process, but the same derivation holds for the semi-Markov process. It is then straightforward to show that excess entropy is

(15) $$E=H\left[X\right]+\sum_{x}p\left(x\right)E\left[\phi_{x}\left(t\right)\right]$$

where

(16) $$E\left[\phi_{x}\left(t\right)\right]=\int_{0}^{\infty }\int_{0}^{\infty }\mu_{x}\phi_{x}\left(t+t^{\prime }\right)\log\frac{\phi_{x}\left(t+t^{\prime }\right)}{\mu_{x}\Phi_{x}\left(t\right)\Phi_{x}\left(t^{\prime }\right)}dtdt^{\prime }.$$

Figure 5b shows how excess entropy E varies with $f_{T},\;b_{T}$. Interestingly, E varies in opposition to $C_{\mu }$, attaining its lowest values at low values of $f_{T}$ and $b_{T}$. Hence, the singular limits $f_{T},\;b_{T}\to 0$ that plague $C_{\mu }$ are not singular limits for E. Nor are $f_{T}^{\prime }\;,b_{T}^{\prime }\to 0$ singular limits for E, as arbitrarily small values of $f_{T}^{\prime }\;,b_{T}^{\prime }$ lead to arbitrarily small perturbations to the trajectory distribution, and thus arbitrarily small perturbations to the mutual information between past and future.

How different would the results be for n>1, i.e., when the number of binding sites of the MWC molecule exceeded 1? In short, we would expect the same qualitative trends and singular limits. In this more general case, we allow an active MWC molecule with *k* ligands bound to transition to an inactive MWC molecule with *k* ligands bound, and vice versa, both with rates $f_{T}^{\prime }$ and $b_{T}^{\prime }$, as for n=1. Meanwhile, also as for n=1, the active MWC molecule with no ligands bound can transition to the inactive MWC molecule with no ligands bound with rate $f_{T}$, and the reverse transition can occur with rate $b_{T}$. The observed process for fixed ligand concentration would still be semi-Markov when $f_{T}^{\prime }=b_{T}^{\prime }=0$, as was true for n=1. Then, decreases in $f_{T},\;b_{T}$ would lead to longer dwell times in active and inactive states, thereby increasing the statistical complexity $C_{\mu }$; and the dwell time distributions would become closer to exponential, decreasing the excess entropy E. When $f_{T}^{\prime },\;b_{T}^{\prime }$ become small but nonzero, all pasts are causal states, and so $C_{\mu }$ shoots to infinity, while E (because it is a function of trajectory distributions) barely changes.

There are some well-known examples of how arbitrarily large ϵ-machines can still have arbitrarily small excess entropies, e.g., the almost fair coin. Indeed, Ref. [23] defined crypticity as the difference between statistical complexity $C_{\mu }$ and excess entropy E. The dynamical MWC molecule described above adds another such example to the literature, finding not only that a familiar process can have arbitrarily large crypticity, but that $C_{\mu }$ and E can be anti-correlated with respect to underlying kinetic rates, as is true for the process generated by the parametrized Simple Nonunifilar Source [25]. There are also examples in the literature of processes with uncountable ϵ-machines and nonzero box-counting dimensions of their mixed-state presentation, e.g., the Cantor process in Ref. [26].

However, the dynamical MWC molecule is more than just an example of a process with potentially arbitrarily large crypticity or an uncountable ϵ-machine; it is also an example of how arbitrarily small changes to a generative model can lead to arbitrarily large changes in the causal structure of a process. Of course, it may be obvious to those familiar with intrinsic computation that sometimes, arbitrarily small perturbations in transition probabilities of a generative model can lead to arbitrarily large perturbations in ϵ-machine structure. However, to the author’s knowledge, the above MWC molecule example is the first such example in the literature.

### 3.2. Functional Computation

Monod-Wyman-Changeux (MWC) molecules have been used to model everything from ligand-gated ion channels to gene regulation [8]. The functional computations that an MWC molecule is thought to perform include transduction of ligand concentration and low-pass filtering of input [7].

**Figure 6:**
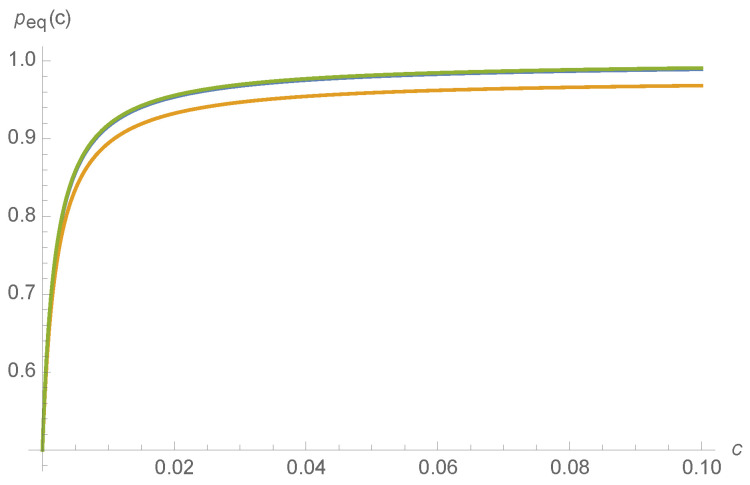
Probability of being in the active state, $p_{eq,A}\left(c\right)$, as a function of ligand concentration *c*, for $f_{T}=1,\;f_{A}=100,\;b_{A}=0.1$ and $b_{T}=1,\;f_{I}=1,\;b_{I}=1$, and: $f_{T}^{\prime }=b_{T}^{\prime }=0$ (blue); $f_{T}^{\prime }=0.01$ and $b_{T}^{\prime }=0$ (orange); and $f_{T}^{\prime }=0$ and $b_{T}^{\prime }=10$ (green), almost overlaying the blue.

Let ${\mathrm{eig}}_{0}\left(M\left(c\right)\right)$ be the normalized eigenvector of eigenvalue 0 of matrix M(c), normalized so that $1^{⊤}{\mathrm{eig}}_{0}\left(M\left(c\right)\right)=1$; and let $p_{eq,A}\left(c\right)$ be the equilibrium probability of being in state *A*. The MWC molecule’s ability to convey the ligand concentration via its activity is a static property, relying only on how the equilibrium distribution

(17) $$\begin{array}{ccc} p_{eq,A}\left(c\right) & = & p_{eq,A_{0}}\left(c\right)+p_{eq,A_{1}}\left(c\right)\\ & = & {\left({\hat{e}}_{1}+{\hat{e}}_{2}\right)}^{⊤}{\mathrm{eig}}_{0}\left(M\left(c\right)\right) \end{array}$$

varies with kinetic rates. An observer that can only see whether or not the MWC molecule is active can discern, to some extent, the external ligand concentration *c*. Such a situation might occur, for instance, for the nicotinic acetylcholine receptors at the neuromuscular junction that transduce information about whether or not a muscle fiber should seize, based on acetylcholine concentration. Though ${\mathrm{eig}}_{0}\left(M\left(c\right)\right)$ in principle might be a non-smoothly varying function of $f_{T}^{\prime },\;b_{T}^{\prime }$, a Mathematica calculation finds that ${\mathrm{eig}}_{0}\left(M\left(c\right)\right)$ and thus $p_{eq,A}\left(c\right)$ varies smoothly with kinetic rates $f_{T}^{\prime },\;b_{T}^{\prime }$:

$${\mathrm{eig}}_{0}\left(M\left(c\right)\right)\propto \left(\begin{array}{c} b_{A}b_{I}b_{T}+b_{A}b_{T}b_{T}^{\prime }+b_{A}b_{T}^{\prime }f_{I}c+b_{I}b_{T}f_{T}^{\prime }\\ (b_{I}b_{T}f_{A}+b_{T}b_{T}^{\prime }f_{A}+b_{T}^{\prime }f_{A}f_{I}c+b_{T}^{\prime }f_{T}f_{I})c\\ b_{A}b_{I}f_{T}+b_{A}f_{T}b_{T}^{\prime }+f_{T}^{\prime }b_{I}f_{A}c+b_{I}f_{T}f_{T}^{\prime }\\ (b_{A}f_{T}f_{I}+b_{T}f_{A}f_{T}^{\prime }+f_{T}^{\prime }f_{I}f_{A}c+f_{T}^{\prime }f_{T}f_{I})c \end{array}\right),$$

where the normalization constant is chosen so that $1^{⊤}{\mathrm{eig}}_{0}\left(M\left(c\right)\right)=1$. The smooth variation of $p_{eq,A}\left(c\right)$ with respect to $f_{T}^{\prime },\;b_{T}^{\prime }$ is depicted for a random choice of kinetic rates in Figure 6.

The MWC molecule is a low-pass filter of ligand concentration *c*. Suppose that $c\left(t\right)=c_{0}+\delta c\;\sin\omega t$, where δc is small. Then $p_{eq,A}\left(t\right)$ will also take the form $p_{eq,A}=p_{eq,A}\left(c_{0}\right)+G\left(\omega \right)\delta c\;\sin\omega t+O\left(\delta c^{2}\right)$, where G(ω) is the transfer function. This transfer function therefore characterizes the dynamical response of the MWC molecule to fluctuations in the ligand concentration. From Equation 49 of Ref. [8], we find that the transfer function G(ω) is

(18) $$G\left(\omega \right)={\left({\hat{e}}_{1}+{\hat{e}}_{2}\right)}^{⊤}{\left(i\omega I-M_{0}\right)}^{-1}M_{1}{\mathrm{eig}}_{0}\left(M\left(c_{0}\right)\right)$$

where

$$\begin{array}{ccc} M_{0} & = & \left(\begin{array}{cccc} -f_{T} & b_{A} & b_{T} & 0\\ 0 & -(f_{T}^{\prime }+b_{A}) & 0 & b_{T}^{\prime }\\ f_{T} & 0 & -b_{T} & b_{I}\\ 0 & f_{T}^{\prime } & 0 & -(b_{T}^{\prime }+b_{I}) \end{array}\right)\mathrm{and}\\ M_{1} & = & \left(\begin{array}{cccc} -f_{A} & 0 & 0 & 0\\ f_{A} & 0 & 0 & 0\\ 0 & 0 & -f_{I} & 0\\ 0 & 0 & f_{I} & 0 \end{array}\right). \end{array}$$

**Figure 7:**
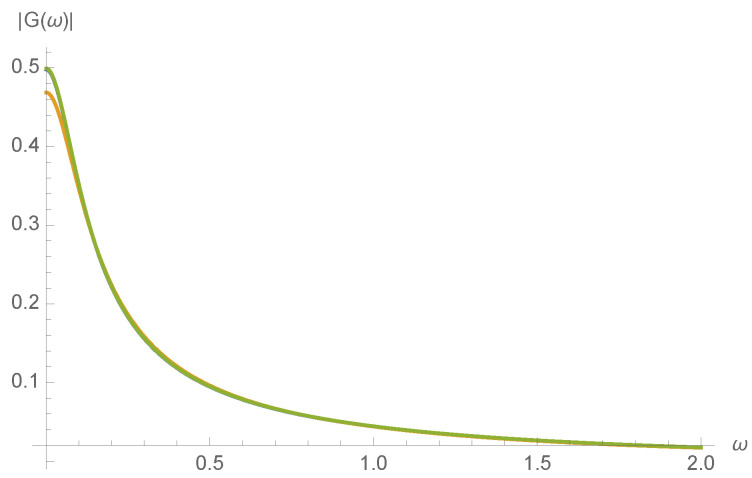
Transfer function G(ω) as a function of input frequency ω at randomly chosen initial concentration $c_{0}$ for $f_{T}=1,\;f_{A}=100,\;b_{A}=0.1$ and $b_{T}=1,\;f_{I}=1,\;b_{I}=1$, and: $f_{T}^{\prime }=b_{T}^{\prime }=0$ (blue); $f_{T}^{\prime }=0.01$ and $b_{T}^{\prime }=0$ (orange); and $f_{T}^{\prime }=0$ and $b_{T}^{\prime }=10$ (green), almost overlaying the blue.

A series expansion not shown here confirms that G(ω) varies smoothly with kinetic rates $f_{T}^{\prime },\;b_{T}^{\prime }$, as would be expected from the realization that all expressions in Equation (18) are smoothly varying with $f_{T}^{\prime },\;b_{T}^{\prime }$. To illustrate this, the magnitude of the transfer function, |G(ω)|, is plotted in Figure 7 for a randomly chosen initial concentration of $c_{0}=1$.

Again, it is worth commenting on how these results would vary with larger *n*, i.e., a larger number of potential ligands bound to the MWC molecule. We consider the dynamical model for this more complex MWC molecule as specified in Section 3.1. Just as for the case when n=1, the eigenvector of eigenvalue 0 for this larger MWC molecule’s rate matrix is a continuous function of kinetic rates $f_{T},\;b_{T}$ and $f_{T}^{\prime },\;b_{T}^{\prime }$; as a result, both the binding curve and the transfer function vary smoothly with these rates.

## 4. Discussion

Our overarching aim here was to study the link between intrinsic computation and functional computation by focusing on a popular model of a biological sensor–the Monod-Wyman-Changeux (MWC) molecule. While studying its intrinsic computation, we found interesting singular limits for $C_{\mu }$. In particular, we found that statistical complexity was infinite and that all pasts were causal states when two of the kinetic rates were nonzero, $f_{T}^{\prime },\;b_{T}^{\prime }\neq 0$, no matter how small $f_{T}^{\prime },\;b_{T}^{\prime }$; and we found that statistical complexity was zero when $f_{T}=b_{T}=0$ but nonzero and arbitrarily large for arbitrarily small $f_{T},\;b_{T}>0$. While studying the MWC molecule’s functional computation and its process’ excess entropy E [10,11,12], we found no such singular limits with respect to these kinetic rates.

The reason for this is that the studied functional computations and excess entropy is a continuous function of trajectory distributions alone, while statistical complexity must be written in terms of a distribution of causal states, which can vary in a non-continuous manner with the trajectory distribution. As a result, statistical complexity (and the mixed state presentation’s box-counting dimension $h_{0}$) and the ϵ-machine are incredibly sensitive to a particular type of process structure, which includes but is not limited to forbidden words. On the other hand, the studied functional computations and excess entropy are smoothly varying functions of the generative model’s kinetic rates.

From the study of the MWC molecule alone, we can conclude that a restrictive definition of intrinsic computation, e.g., only causal structure, does not necessarily provide a guide to the functional computation of a dynamical system, at least for the functional computations considered here. Accordingly, high statistical complexity does not imply biological function.

It’s well worth emphasizing that the functional computations listed here are far from an exhaustive list of all possible functional computations, and so future research might uncover a functional computation that depends sensitively on the process’ causal structure. Also, even if no such functional computation is identified, the sensitivity of causal structure to certain changes in the generative model might be considered by some to be an interesting feature, and not a bug, perhaps as a case study in how limited computational resources yield innovation [1]. However, for those wishing to study functional computation only, the extreme sensitivity of statistical complexity to particular types of process structure might prove to be a bug rather than a feature.

However, even then, the ϵ-machine finds use. In more recent years, intrinsic computation has been expansively defined to include a study of other structure-related information-theoretic statistics of a process besides statistical complexity [21]. This list includes but is not limited to excess entropy [10,11,12] as studied here, bound information rate [27], and predictive rate-distortion functions [28,29]. The last is particularly notable here in that predictive rate-distortion includes statistical complexity and excess entropy as limiting cases. On the whole, these quantities enjoy the label “information anatomy” [19] or the more broadly-construed “informational architecture”. Most of these quantities are smoothly varying functions of the transition probabilities in the minimal generative model and thus trajectory distribution, and so are not as sensitive to the underlying structure of the process as is statistical complexity. (The statistical complexity can vary discontinuously with transition probabilities in the minimal generative model, but varies smoothly with the transition probabilities in the minimal maximally predictive model; the structure of the minimal maximally predictive model, the ϵ-machine, can change by an arbitrarily large amount with arbitrarily small changes to the minimal generative model.) Those that are not so sensitive to the process’ structure are often easily calculable from the process’ ϵ-machine [29,30]. In the future, these quantities might provide interesting statistics with which to interpret the functional computation performed by biological or social systems, e.g., as in Ref. [31].
